# ELDER MISTREATMENT AND RELATIONSHIP QUALITY: SECONDARY DATA ANALYSIS USING DATA FROM THE NSHAP

**DOI:** 10.1093/geroni/igac059.2434

**Published:** 2022-12-20

**Authors:** Wei-Lin Xue, Zach Hass, Pi Ju Liu

**Affiliations:** Purdue University, Lafayette, Indiana, United States; Purdue University, West Lafayette, Indiana, United States; Purdue University, Lafayette, Indiana, United States

## Abstract

As social networks shrink with age, older adults value the importance of interpersonal relationships with close others, such as partners, family and friends. Previous studies focused on perpetrators' and victims' characteristics; however, few studies examined the relationships with close others and the incidence of elder mistreatment. This study used the National Social Life, Health, and Aging Project Wave 1 data (2005-06) to examine the correlations between relationship quality with close others and the occurrence of mistreatment among older adults (N = 3005). Based on spousal relationship literature using the NSHAP data, factor analysis was used to estimate factor scores conceptualizing two domains of relationship quality: relationship support (positive dimensions of relationship), and relationship strain (negative dimensions of the relationship). Logistic regression models were used to test the relationship between the relationship quality factor scores and the likelihood of each mistreatment type while controlling for gender, education, age, and race. Psychological abuse was more likely for older adults experiencing relationship strain with spouse (OR=1.82, p<.001), family (OR=1.75, p<.001), and/or friends (OR=1.67, p< 0.001). Financial abuse was more likely for those experiencing poor relationship support with family (OR=1.34, p<.05) and those experiencing relationship strain with friends (OR=1.47, p<.01). However, relationship quality was not correlated with likelihood of physical abuse. Interpersonal relationships with close others could provide stronger support and care, but relationship strain might contribute to mistreatment. When determining safeguarding gatekeepers to protect older adults from potential mistreatment, interventions should consider the quality and composition of interpersonal relationships with close others.

